# The role of microorganisms on biotransformation of brewers’ spent grain

**DOI:** 10.1007/s00253-020-10843-1

**Published:** 2020-09-02

**Authors:** Angela Bianco, Marilena Budroni, Severino Zara, Ilaria Mannazzu, Francesco Fancello, Giacomo Zara

**Affiliations:** Department of Agricultural Science, University of Sassari, Sassari, Italy

**Keywords:** Microbial communities, Microbial quality, Mycotoxins, Single-cell protein, Vermicompost, Brewers’ spent grain

## Abstract

Brewers’ spent grain (BSG) is the most abundant by-product of brewing. Due to its microbiological instability and high perishability, fresh BSG is currently disposed of as low-cost cattle feed. However, BSG is an appealing source of nutrients to obtain products with high added value through microbial-based transformation. As such, BSG could become a potential source of income for the brewery itself. While recent studies have covered the relevance of BSG chemical composition in detail, this review aims to underline the importance of microorganisms from the stabilization/contamination of fresh BSG to its biotechnological exploitation. Indeed, the evaluation of BSG-associated microorganisms, which include yeast, fungi, and bacteria, can allow their safe use and the best methods for their exploitation. This bibliographical examination is particularly focused on the role of microorganisms in BSG exploitation to (1) produce enzymes and metabolites of industrial interest, (2) supplement human and animal diets, and (3) improve soil fertility. Emerging safety issues in the use of BSG as a food and feed additive is also considered, particularly considering the presence of mycotoxins.

**Key points**

• *Microorganisms are used to enhance brewers’ spent grain nutritional value.*

• *Knowledge of brewers’ spent grain microbiota allows the reduction of health risks.*

**Figure:**
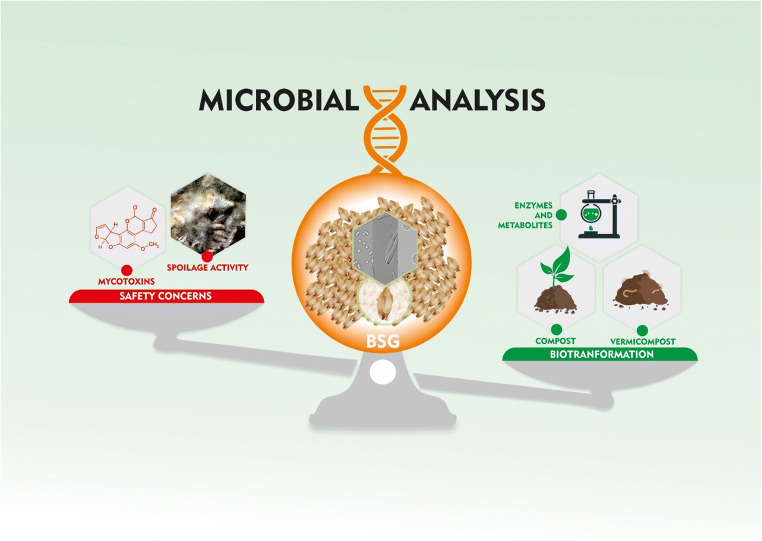
Graphical abstract

Graphical abstract

## Introduction

**Fig. 1:**
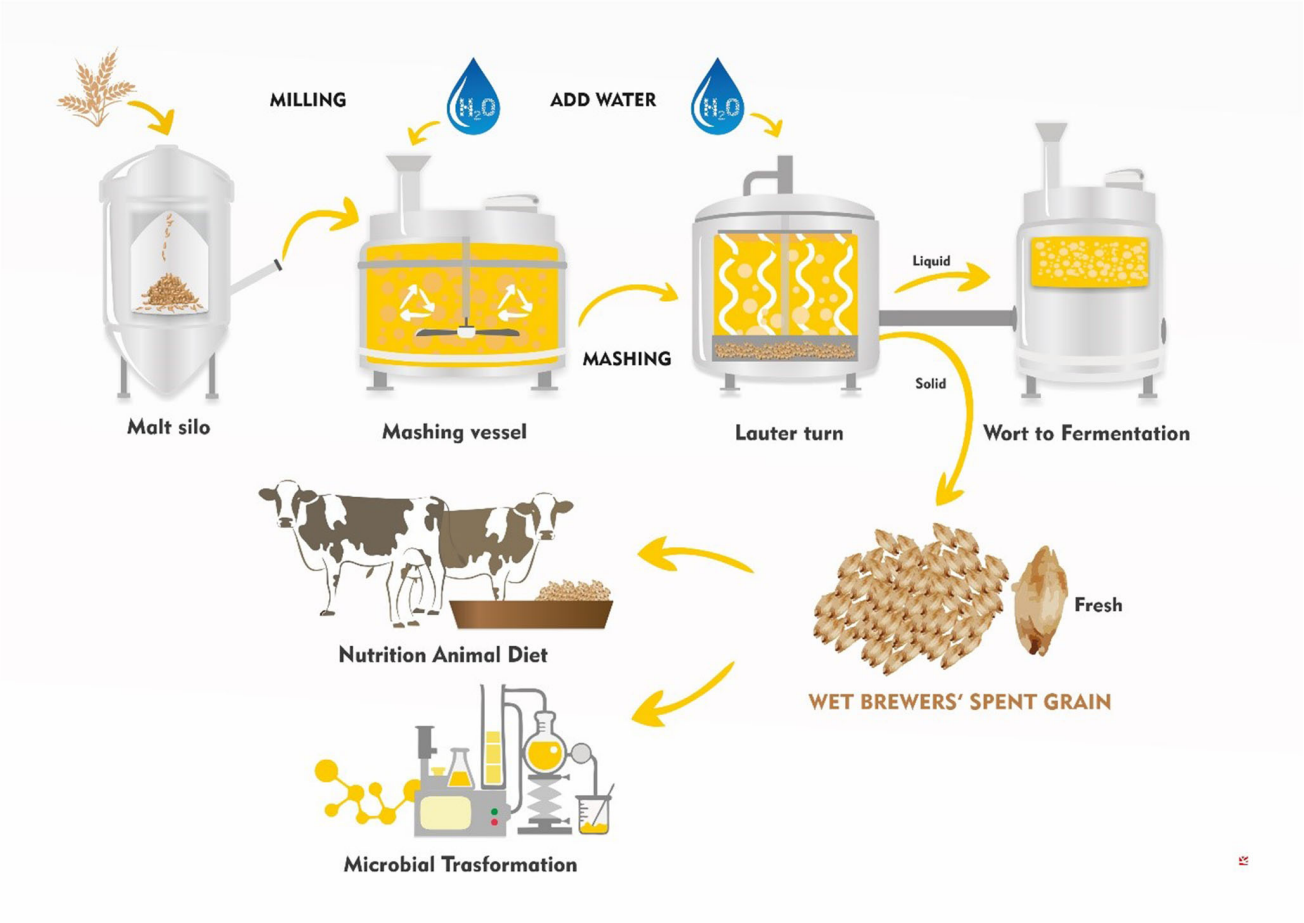
Scheme of the brewing process and consecutive production of brewers’ spent grain

The recycling and exploitation of brewing residues, such as brewery wastewater, surplus yeast, and brewers’ spent grain (BSG), are critical goals for reduction of energy consumption and residue disposal costs, and also for reduction of the associated carbon foot-print (Zupančič et al. 2017). BSG is the most abundant by-product from the brewing process (i.e., 85% of total by-products) (Fig. 1). Some 100 to 130 kg of fresh BSG (humidity, 70–80%) are obtained from 100 kg of malt, which also equates to 21 to 22 kg BSG per hectoliter of beer brewed (Kunze 2004). The global production of BSG has reached 39 million tons per year on average (Birsan et al. 2019). Of this, 3.4 million tons are produced in Europe, which ranges from 2 million tons/year in Germany (Steiner et al. 2015) to 288,000 tons/year in Italy (Assobirra Annual Report 2018). Around 70% of the BSG produced is used as animal feed, with 10% used to produce biogas and the remaining 20% disposed of as landfill. Its relatively low cost makes BSG an interesting raw material that has the potential to be used for the production of goods with high added value.

**Fig. 2:**
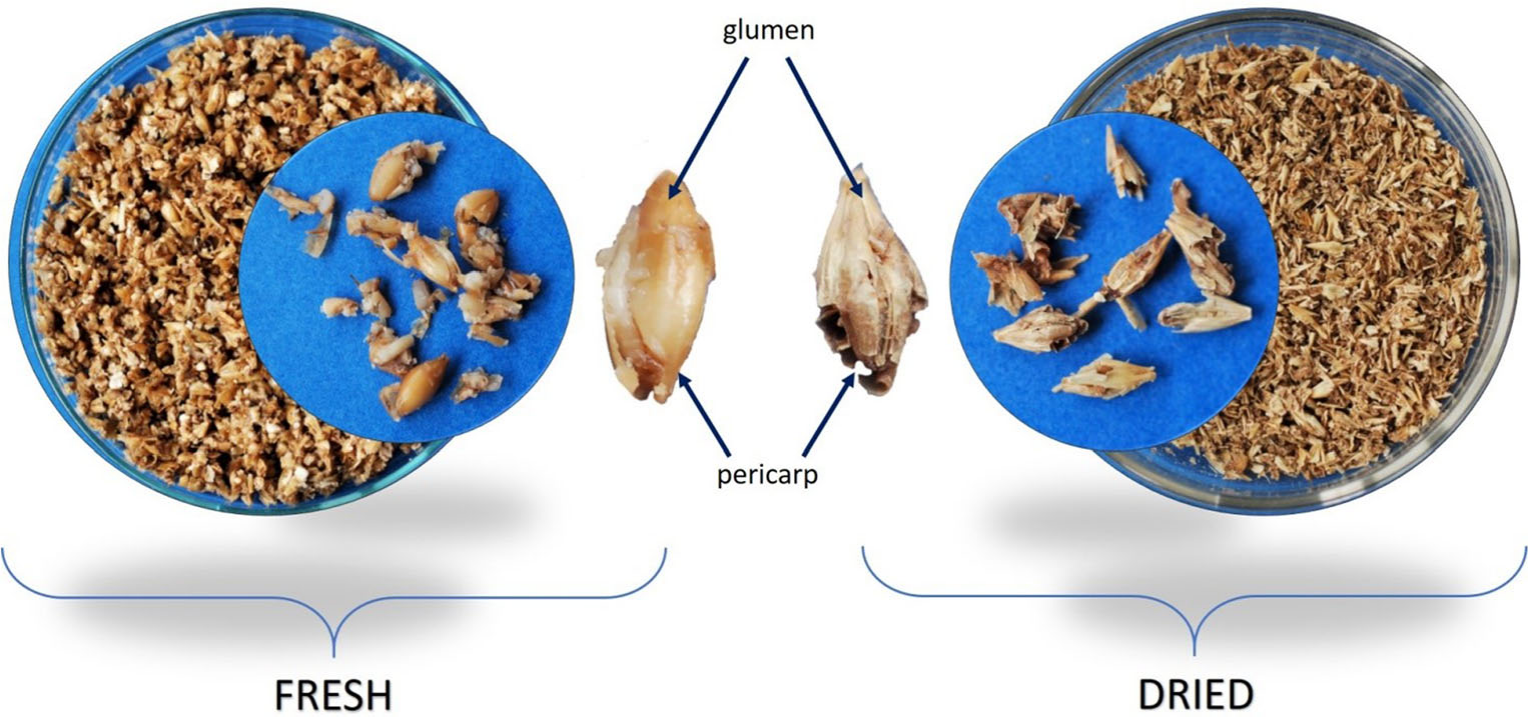
Brewers’ spent grain fresh from production and after drying

Brewers’ spent grain comprises the glumes, pericarp, and integuments of the outer layers of the barley kernels and of the other cereals, which contain nutrients that are not extracted during the malting and mashing processes (Fig. 2) (dos Santos Mathias et al. 2014). The chemical composition of BSG (Table 1) varies depending on barley cultivar, malting process, and quality and formulation of the brewing cereals (Gupta et al. 2010; Santos et al. 2003). Notwithstanding, BSG composition always includes high levels of dietary fiber, protein, and essential amino acids, as well as appreciable levels of minerals, polyphenols, vitamins, and lipids (Fărcaş et al. 2014). The high polysaccharide, protein, and moisture content of BSG makes it susceptible to microbial deterioration over a short period of time (i.e., from 2 to 7 days) (Wang et al. 2014; Gupta et al. 2013).

**Table 1:** Chemical composition of brewers’spent grain

**Group**	**Units**	**Compound**	**Content**
Major components	g kg^−1^ dry weight	Cellulose (glucan)	3–330
		Hemicellulose	192–419
		Xylan	136–206
		Arabinan	56–419
		Starch	10–120
		Lignin	115–278
		Lipids	30–106
		Proteins	142–310
		Ashes	11–46
		Extractives	58–107
		Phenolics	7–20
		Ferulic acid	1860–1948
		*p*-Coumaric acid	565–794
Minerals	mg kg^−1^ dry weight	Silicon	1400–10,740
		Phosphorus	4600–6000
		Calcium	2200–3515
		Magnesium	1900–2400
		Sulfur	1980–2900
		Potassium	258.1–700.0
		Sodium	100.0–309.3
		Iron	100.0–193.4
		Zinc	82.1–178.0
		Aluminum	36.0–81.2
		Manganese	40.9–51.4
		Cobalt	17.8
		Copper	11.4–18.0
		Strontium	10.4–12.7
		Iodine	11
		Barium	8.6–13.6
		Chromium	<0.5–5.9
		Molybdenum	1.4
		Boron	3.2
Non-essential amino acids	% total protein	Histidine	26.27
		Glutamic acid	16.59
		Aspartic acid	4.81
		Valine	4.61
		Arginine	4.51
		Alanine	4.12
		Serine	3.77
		Tyrosine	2.57
		Glycine	1.74
		Asparagine	1.47
		Glutamine	0.07
Essential amino acids	% total protein	Lysine	14.31
		Leucine	6.12
		Phenylalanine	4.64
		Isoleucine	3.31
		Threonine	0.71
		Tryptophan	0.14
Vitamins	ppm	Biotin	0.1
		Choline	1800
		Folic acid	0.2
		Niacin	44
		Phantotenic acid	8.5
		Riboflavin	1.5
		Thiamine	0.7
		Pyridoxine	0.7

Currently, the main solution for the exploitation of BSG continues to be its supply to local farmers for livestock feed. However, BSG production often exceeds the demand for local feed, which results in disposal issues in terms of the sustainability and environmental impact of breweries (Mussatto 2014). Research into new conservation systems and new uses and enhancement technologies, together with better understanding of the use of microorganisms as biocatalysts, are important in the search for new ways to exploit BSG in foods and animal feed, and in the pharmaceutical and cosmetic sectors. Consequently, patents have been filed recently in connection with BSG (Table 2), while others were originally filed from 1965.

**Table 2:** Patents held for brewers’ spent grain

**Title of patent**	**Number**	**Source**	**Brief description**	**References, Inventor, Year**
Process for producing a filler from brewer’s spent grain (BSG), filler, use of a filler and foodstuff	US Patent Application: US-2020-0138065-A1	http://www.freepatentsonline.com/y2020/0138065.html	A process for producing a filler from BSG. The process comprises (1) comminuting the BSG; (2) heating the BSG; (3) optionally, fermenting the BSG; (4) optionally, reducing the moisture content of the BSG; and (5) optionally, mixing the BSG with at least one flavor-modifying food additive, such as a sugar substitute(s) and/or an aroma. Also disclosed are a filler obtained or obtainable by this process, the use of such a filler, and a foodstuff comprising at least one such filler	Petry and Olwal (2020)
Intermittent infrared drying for BSG	US Patent Application: US-105-78,358-B2	http://patft.uspto.gov/netacgi/nph-Parser?Sect1=PTO1&Sect2=HITOFF&p=1&u=/netahtml/PTO/srchnum.html&r=1&f=G&l=50&d=PALL&s1=10578358.PN	A system for processing BSG that includes a specific intermittent infrared heating and stirring protocol designed to produce a unique dried BSG product that can be used whole or ground up and used as a quality flour suitable for human consumption	McHugh et al. (2020)
Method for reducing moisture in BSG	US Patent Application: US-2019-0254315-A1	http://appft.uspto.gov/netacgi/nph-Parser?Sect1=PTO1&Sect2=HITOFF&p=1&u=/netahtml/PTO/srchnum.html&r=1&f=G&l=50&d=PG01&s1=20190254315.PGNR	A method for reducing the moisture content of BSG comprising the exposure of different BSG to infrared radiation	Willis (2019)
Process for a prepared beverage or beverage component from BSG	US Patent Application: US-2019-0200640-A1	http://appft.uspto.gov/netacgi/nph-Parser?Sect1=PTO1&Sect2=HITOFF&p=1&u=/netahtml/PTO/srchnum.html&r=1&f=G&l=50&d=PG01&s1=20190200640.PGNR	A process for preparing a beverage or a component of a beverage. Enzymatic treatment of BSG with addition of one or more enzymes, with α-amylase, gluco-amylase, cellulase, xylanase, protease, and/or β-glucanase activities, and subsequent fermentation with a strain of lactic acid bacteria	Gil-Martinez and Arendt (2019a)
Spent grain fuel product and process	US Patent Application: US-10-364-400-B2	http://patft.uspto.gov/netacgi/nph-Parser?Sect1=PTO1&Sect2=HITOFF&p=1&u=/netahtml/PTO/srchnum.html&r=1&f=G&l=50&d=PALL&s1=10364400.PN	A process of making a fuel product from BSG. After production, the dried BSG is fed into a combustion chamber of a steam boiler that is used for beer brewing, and the BSG is separated during combustion by agitation, such as spraying of the BSG in the combustion chamber	Larson et al. (2019)
A process for microbiological stabilization of BSG, microbiologically stabilized BSG, and the use thereof	Patent Cooperation Treaty: WIPO (PCT); WO-2019-034567-A1	http://patentscope.wipo.int/search/en/WO2019034567	A process to microbiologically stabilize the spent fresh beer beans (BSG). The process includes acidification of the BSG to a pH 4 or lower, on the basis that the BSG is acidified before reaching mycotoxin levels above the limits of reads and/or counts of colonies, not exceeding 10^3^ CFU g^−1^ aerobic bacteria, fungi, yeast, mesophilic aerobic bacteria, or total anaerobic bacteria, after a week of storage at 25 °C	Gil-Martinez and Arendt (2019b)
Composition of BSG and polylactic acid	US Patent Application: US-10-285,422-B2	http://patft.uspto.gov/netacgi/nph-Parser?Sect1=PTO1&Sect2=HITOFF&p=1&u=/netahtml/PTO/srchnum.html&r=1&f=G&l=50&d=PALL&s1=10285422.PN.	A composition consisting of BSG and polylactic acid (PLA) is produced by the following steps: processing BSG; mixing BSG with PLA at a specific proportion; BSG and PLA are well mixed by a binder and then subjected to a granulation process to form plastic granules. Adding BSG to PLA can reduce the use of PLA. Furthermore, the composition of BSG and PLA and the method for making this are further used to make utensils, bottles, cans, containers, parts, and other biological plastic products, to provide added value to BSG	Chen et al. (2019a, b)
Composition consisting of BSG and PLA and a method for making the same	US Patent Application: US-10-201,177-B2	http://patft.uspto.gov/netacgi/nph-Parser?Sect1=PTO1&Sect2=HITOFF&p=1&u=/netahtml/PTO/srchnum.html&r=1&f=G&l=50&d=PALL&s1=10201177.PN	This method for manufacturing a composite consisting of BSG and PLA includes the steps of: providing the raw material containing BSG; providing the raw material containing PLA; mixing the BSG with the PLA at a specific proportion; and providing a binder to enable the BSG and the PLA to be well mixed, and to maintain the desired tensile strength. A pretreatment unit includes dehydration, desiccation, drying, grinding, and sieving. The granulator includes a double screw extruder connected to a cutting machine. The double screw extruder mixes the BSG and the PLA and extrudes them into plastic bars that are then cut into plastic granules by the cutting machine	Chen et al. (2019a, b)
Systems and methods for making BSG dough products	US Patent Application: US-2019-0223457-A1	http://appft.uspto.gov/netacgi/nph-Parser?Sect1=PTO1&Sect2=HITOFF&p=1&u=/netahtml/PTO/srchnum.html&r=1&f=G&l=50&d=PG01&s1=20190223457.PGNR	The invention provides methods to produce dough and bread products made at least in part of BSG. The BSG is removed from the brewing or distilling process, dried, frozen, and then further processed into a BSG dough product. More specifically, the invention relates to the systems and methods for making frozen pizza dough balls composed at least in part of grain products from the process of brewing beer or distilling sprits, such as whiskey	Brown and Allgeier (2019)
Process for producing protein concentrate or isolate a cellulosic thermochemical feedstock from BSG	US Patent Application: US-2018-0199593-A1 US-2018-0199594-A1 European Patent Application: WO-2018-136,235-A1, WO-2018-136,234-A1	https://data.epo.org/publication-server/search http://www.freepatentsonline.com/y2018/0199593.html	A process to obtain a high-value protein product and a cellulosic residue from BSG. The high-value protein product is useful as a protein supplement or as feed for livestock and poultry, and the cellulose residue has value as a raw material for a thermochemical process unit, to produce a biofuel	Mackay and Greden (2018)
BSG-based protein powder	US Patent Application: US-2018-0014555-A1	http://appft.uspto.gov/netacgi/nph-Parser?Sect1=PTO1&Sect2=HITOFF&p=1&u=/netahtml/PTO/srchnum.html&r=1&f=G&l=50&d=PG01&s1=20180014555.PGNR	Production of a BSG-based protein powder and the related methods for using the powder in protein-enriched foods. The BSG-based protein powders are highly soluble and therefore easily wettable, easily dispersible, and mixable at concentrations up to 50% by weight. They can be used alone or as a protein enhancer in food for human consumption, pet food, and commercial feed	Mackay et al. (2018)
Bio-plastic composite containing BSG and a method for making the same	US Patent Application: US-2017-0306153-A1 US-10-030-148-B2	http://patft.uspto.gov/netacgi/nph-Parser?Sect1=PTO1&Sect2=HITOFF&p=1&u=/netahtml/PTO/srchnum.html&r=1&f=G&l=50&d=PALL&s1=10030148.PN	Method for obtaining a bioplastic composite from BSG	Chen et al. (2018)
Integrated process for extracting proteins and arabinoxylans from BSG	Patent Cooperation Treaty: WIPO (PCT); WO-2012-069889-A1	https://worldwide.espacenet.com/patent/search/family/044479068/publication/WO2012069889A1?q=pn%3DWO2012069889A1	The present invention proposes an integrated process for extracting proteins and arabinoxylans from BSG, without the need to subject the BSG to any pre-treatment, through the use of alkaline reagents followed by selective precipitation by acidification of the medium and addition of ethanol. The present invention is applicable in the areas of re-use or exploitation of BSG, with the aim to obtain products that can be used as ingredients in the food industry, and in the production of dietetic and pharmaceutical products. The final residue obtained after extraction of the proteins and arabinoxylans can be used as a source of cellulose, as an insoluble dietetic fiber, or possibly as a fuel or raw material for the paper-making industry	Saraiva et al. (2012)
Integrated process of filtration to dry BSG	Patent Cooperation Treaty (PCT): WO-2010-117,288-Al WO-2010-117,288-A8	https://worldwide.espacenet.com/patent/search/family/042026531/publication/WO2010117288A1?q=pn%3DWO2010117288A1	A process and the corresponding equipment, to dehydrate BSG from 85 to 15% humidity, to obtain a stabilized product with the same content of protein, fiber, and lipids. The dehydration process involves several phases, two of which are mechanical (membrane filtration, compression) and the last one of which consists of vacuum drying using hot water or low-pressure water vapor as a heat source. Energy for the process is available at no cost in the brewing industry, through the use of hot process water or low-pressure steam from cogeneration units. Dehydrated BSG is a stabilized product and can be used as food for humans and animals, ruminants, and non-ruminants, and as a raw material for biotechnological and pharmaceutical applications	De Carvalho et al. (2010)
Process for drying BSG	US Patent Application: US-2012-0005916-A1 Patent Cooperation Treaty (PCT): WO-2010-053493-Al	http://appft.uspto.gov/netacgi/nph-Parser?Sect1=PTO1&Sect2=HITOFF&p=1&u=/netahtml/PTO/srchnum.html&r=1&f=G&l=50&d=PG01&s1=20120005916.PGNR	A process for drying BSG, to obtain a product with a moisture content ≤ 15% by weight, biologically stable over time, with high nutritional value, commercially profitable, and environmentally safe, comprising two phases: the first is a mechanical pressing operation to reduce the initial humidity of the BSG by at least 75–80% by weight, up to a humidity < 70% by weight; the liquid obtained is transported to an effluent treatment plant; the solid obtained undergoes a second phase that consists of thermal drying, with two sub-phases: during the first, the product is transported through a stream of hot air, while during the second, it is transported by an air flow at room temperature	Lopez et al. (2012)

In recent years, different aspects related to the chemical composition of BSG have been reviewed, from its use as a promising integrated feed source of prebiotic compounds for livestock (Lao et al. 2020) and a concentrate feed for lactating dairy cows (Chanie and Fievez 2017), to the best extraction methods to preserve its bio-active compounds (Bonifácio-Lopes et al. 2019; Guido and Moreira 2017). In particular, the antioxidant, anti-atherogenic, anti-inflammatory, and anti-carcinogenic activities of chemical compounds in BSG have been considered (Ikram et al. 2017; Lynch et al. 2016; McCarthy et al. 2013). Finally, methods for exploitation of BSG in terms of food and energy production, and in the chemical and agronomic sectors have been reviewed (Cancelliere et al. 2019; Mussatto 2014; Xiros and Christakopoulos 2012; Aliyu and Bala 2011; Mussatto et al. 2006). In this context, the aim of this mini-review is to present the current literature on BSG from a microbiological point of view. Indeed, the knowledge of different BSG-related microbiological aspects is still limited, particularly when compared with the above-cited literature regarding the exploitation of its chemical components. However, BSG can support the growth of spoilage microorganisms as well as beneficial microorganisms, and these diverse microorganisms and their interactions can determine the safety aspects and full exploitation of BSG.

## Brewers’ spent grain storage and stabilization

Immediately after lautering, BSG has limited microbial contamination (10^2^–10^3^ CFU g^−1^; Robertson et al. 2010a) and it can be considered as microbiologically stable and within acceptable limits for food use. However, BSG takes several hours to cool down to room temperature (20 °C), and during this period microbial activity continues. The extensive changes in its structure that occur during malting and mashing make it accessible to hydrolytic enzymes, thus making BSG an easier substrate for microbial attack. Consequently, after only 5 days of storage at 20 °C, the microbial concentrations can increase to 10^6^ CFU g^−1^, with microaerophilic, strictly anaerobic, and aerobic, mesophilic, and thermophilic bacteria representing the predominant naturally associated microflora (Robertson et al. 2010a, 2010b). In addition, filamentous fungi are frequently isolated after storage of BSG at room temperature, such as *Aspergillus* spp*.*, *Fusarium* spp., *Mucor* spp., *Penicillium* spp., and *Rhizopus* spp. (Robertson et al. 2010a). Therefore, BSG must be stabilized and stored under the appropriate conditions if it is to be used at a later stage.

Many breweries are discouraged from drying BSG at 60 °C due to the high associated energy costs (Aliyu and Bala 2011; Tang et al. 2009). Robertson et al. (2010b) compared different methods of BSG storage with respect to microbial proliferation, from the fresh material at 20 °C to that refrigerated at 4 °C, autoclaved at 120 °C for 1 h, and frozen. They reported that under storage at 4 °C over 16 days, the numbers of aerobic bacteria in BSG remained < 10^6^ CFU g^−1^, while in the frozen and autoclaved samples, there was no evidence of microbial activity. Indeed, when it was stored at 4 and 20 °C, BSG showed sugars loss that was ascribable to the activities of microbial enzymes, such as xylanases, esterases, and cellulases (Robertson et al. 2010b). Autoclaving was seen as effective for long-term BSG stability (Lynch et al. 2016).

## Brewers’ spent grain in industrial biotechnology

**Fig. 3:**
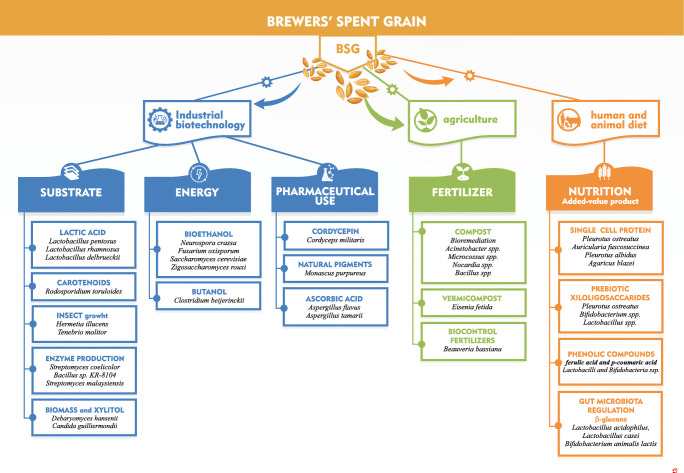
Microbial exploitation of brewers’ spent grain in industrial biotechnology, agricultural processes, and human and animal diets

Brewers’ spent grain is a valuable substrate for microbial growth, and it fulfills some of the requirements demanded for biotechnological exploitation, including regular availability and low market price. Bacteria, fungi, and yeast have been successfully used for the biotechnological exploitation of BSG to produce enzymes and metabolites, microbial biomass, pharmaceuticals, and substrates for biocontrol agents (Fig. 3). BSG has also been used as a substitute to expensive carbon sources for industrial production of lactic acid by *Lactobacillus delbrueckii* UFV H2B20 (Mussatto 2014; Mussatto et al. 2007, 2008), *Lactobacillus pentosus* CECT-4023T, and *Lactobacillus rhamnosus* CECT-288 (Cruz et al. 2007).

Plaza et al. (2017) studied the feasibility of using BSG as a raw material to obtain butanol through an acetone–butanol–ethanol fermentation process using *Clostridium beijerinckii* DSM 6422. Although their results confirmed the suitability of BSG for the production of butanol, further studies are needed to scale-up this process. *Neurospora crassa* DSM 1129 and *Fusarium oxisporum* F3 isolated from cumin were used to convert the cellulose and hemicellulose in BSG directly into bio-ethanol, through successive phases of hydrolysis of the polysaccharides and fermentation of the monosaccharides (Xiros and Christakopoulos 2009; Xiros et al. 2008). The hydrolysis of BSG recalcitrant biopolymers (such as lignin and cellulose) during a pre-fermentation phase carried out by *Aspergillus oryzae* has been shown to improve the availability of nutrients, thus providing the complete requirements for growth of the yeast *Rodosporidium toruloides* (strain CBS 5490), a natural producer of carotenoids (Cooray et al. 2017).

Alternatively, the hydrolysis of BSG can be carried out through alkali, acid, hydrothermal, or enzymatic pre-treatments (Rojas-Chamorro et al. 2020; Paz et al. 2019). Ethanologenic microorganisms have been used to produce ethanol from these hydrolyzed BSG, including *Saccharomyces cerevisiae* and *Zigosaccharomyces rouxii* (Liguori et al. 2015). In particular, the high gravity brewing yeast *S. cerevisiae* BLGII 1762 and *S. cerevisiae* PE-2 isolated from the Brazilian bioethanol industry produce ethanol with yields of between 23 and 81% (Pinheiro et al. 2019). In addition, hemicellulosic hydrolysates of BSG have been used to produce microbial biomass and xylitol using *Debaryomyces hansenii* CCMI 941 (Carvalheiro et al. 2007) and *Candida guilliermondii* FTI 20037 (Mussatto and Roberto 2005). Banjo et al. (2018) used BSG to produce ascorbic acid by submerged fermentation culture of *Aspergillus flavus* and *Aspergillus tamari*, whereby they obtained 6.25 and 7.25 g L^−1^ ascorbic acid, respectively.

Brewers’ spent grain has also been used for isolation and maintenance of actinobacteria, such as *Streptomyces coelicolor* A3 (Szponar et al. 2003) and *S. malaysiensis* AMT-3, and their production of cellulases (Nascimento et al. 2009). *Bacillus* sp. KR-8104 has been grown on BSG to produce α-amylase (Hashemi et al. 2011).

Regarding its exploitation in the pharmaceutical sector, BSG has been used as a culture medium for *Cordyceps militaris* (Gregori 2014) to produce cordycepin, a nucleoside analog with anti-tumor, anti-proliferative, anti-metastatic, insecticidal, and antibacterial activities (Gregori 2014). In addition, polysaccharides from *C. militaris* have shown significant antitumor activities against cervical and liver cancer cells in vitro (Yan et al. 2014), and extracts of its fruiting bodies show antioxidant, antibacterial, antifungal, and anti-tumor activities against human cell lines (Yan et al. 2014; Reis et al. 2013; Rao et al. 2010), and also anti-inflammatory (Rao et al. 2010), anti-fibrotic (Nan et al. 2001), anti-obesity (An et al. 2018), and anti-angiogenetic (Yoo et al. 2004) and insulin-secreting (Choi et al. 2004) activities. Thus, the use of BSG for cordycepin production by *C. militaris* has been shown to be a very effective technique for the production of high-value food and feed additives (Gregori 2014).

In recent years, the food industry has focused on the production of natural pigments from plants and microbial sources to overcome the use of synthetic pigments that can be hazardous to human health and to the environment. Silbir and Goksungur (2019) studied the production of natural red pigments by *Monascus purpureus* strain CMU001 in a submerged fermentation system using BSG as the raw substrate. Pigments from *Monascus* spp. have been used as natural coloring agents and natural food additives in eastern Asia, and have applications as pharmaceuticals, as they have been reported to have anti-mutagenic, anti-cancer, anti-obesity, anti-inflammatory, anti-diabetes, and cholesterol-lowering activities (Silbir and Goksungur 2019).

## Brewers’ spent grain in human and animal diets

The ever-increasing world population and the consequent requests for more food to meet nutritional requirements are two of the factors that define the need for sustainable food production. In particular, the possibility to use waste from agro-industrial processes as raw materials to produce food and feed has drawn the attention of numerous researchers. In this context, BSG provides numerous compounds that can serve as the basis to produce functional food and feed (Bonifácio-Lopes et al. 2019; Skendi et al. 2018). Thus, the use of BSG as a substrate for microbial growth to produce single cell proteins, to enhance its techno-functional properties, and to increase its commercial value has been proposed (Connolly et al. 2019; He et al. 2019; Ibbett et al. 2019; Luft et al. 2019; Marson et al. 2019; Martín-García et al. 2019; Shen et al. 2019).

Single cell proteins are an important example of new healthy proteins with low environmental impact. Their sustainable production represents one of the possible solutions to the problem of protein supply for future generations (Finnigan et al. 2017; Suman et al. 2015). BSG can be used as a low-cost raw material to produce single cell proteins from non-mycotoxigenic filamentous fungi in a sustainable and economic way. Fungi such as *Rhizopus* spp., *Trichoderma* spp., and *Mucor* spp. have been shown to hydrolyze BSG to obtain nitrogen and carbon sources for their growth without the need for costly pre-treatments or enzymatic hydrolysis (Bekatorou et al. 2015).

Brewers’ spent grain has been proposed as a food additive due to its beneficial nutritional properties. These are mainly due to the water-extractable part of BSG, which includes arabinoxylans (Mendis and Simsek 2014) and β-glucans (Steiner et al. 2015) that can regulate the gut microbiota. Possibly due to the release of xylo-oligosaccharides, arabinoxylans promote the growth of *Bifidobacterium* spp. and *Lactobacillus* spp., which have positive effects on the human gastrointestinal tract (Adamberg et al. 2014). Similarly, β-glucans can enhance growth and metabolic activity of beneficial microorganisms, such as *Lactobacillus acidophilus*, *Lactobacillus casei*, and *Bifidobacterium animali subsp. lactis* (Jayachandran et al. 2018). To further stimulate the grow of probiotic microorganisms, BSG has been used as a substrate to cultivate the fungus *Pleurotus ostreatus*, which represents an interesting source of β-glucans with prebiotic activities (Wang et al. 2001). In addition, it has been suggested that the intestinal microbiota, particularly *Lactobacilli* (e.g., *L. salivarius*, *L. paracasei*, *L. rhamnosus*) and *Bifidobacteria* (e.g., *B. adolescentis, B. breve*, *B. longum*), can partially degrade the lignin in BSG and metabolize the compounds released (Niemi et al. 2013). More recently, partial lignin degradation mediated by human fecal microbiota was shown in a colon intestine model (Ohra-aho et al. 2016; Aura et al. 2013). Here, it was suggested that lactic acid bacteria can easily adapt in BSG-based broth, thus paving the way for the use of probiotic strains as starter cultures for further improvement of the bioactive properties of fermented BSG (Gupta et al. 2013).

In addition, phenolic acids of BSG have shown antimicrobial activities against pathogenic bacteria. Ferulic acid is active on *Escherichia coli*, *Klebsiella pneumoniae*, *Enterobacter aerogenes*, *Citrobacter koseri*, *Pseudomonas aeruginosa*, *Helicobacter pylori*, and *Shigella sonnei* (de Oliveira and Batista 2017). *p*-Coumaric acid inhibits several pathogenic bacteria, such as *Staphylococcus aureus 6538*, *Streptococcus pneumoniae* ATCC49619, *Bacillus subtilis* 9372, *E. coli* ATCC25922, *Shigella dysenteriae* 51302, and *Salmonella typhimurium* 50013 (Lou et al. 2012), and it contributes to the definition of the distribution and abundance of microbial populations in the digestive tract (Reverón et al. 2012).

The nutritional value of BSG can be increased through low-cost microbe-mediated transformation processes. Natural degraders of lignocellulosic materials have been reported to significantly increase the nutritional content of BSG by fermentation (e.g., *Aspergillus* spp., *Trichoderma* spp., *Fusarium* spp., *Neurospora* spp.) (Bekatorou et al. 2007). Solid-state fermentation of BSG with *Rhizopus oligosporus* CCT 4134 and *Rhizopus microsporus* var. *oligosporus* (DMSZ1964) successfully increased the levels of amino acids, citric acid, vitamins, and antioxidants, and reduced the levels of carbohydrates, fats, and dietary fiber (Cooray and Chen 2018; Canedo et al. 2016). Further optimization of the operating parameters of solid-state fermentation can significantly increase the protein content, soluble protein, antioxidant and antibacterial activities, and total phenolic content, all of which are characteristics that are highly appreciated for feed and food applications (Ibarruri et al. 2019). *Bacillus subtilis* WX-17 (NCIMB 15204) isolated from a traditional food in Japan has been used for enrichment of the BSG content of triglycerides, palmitic, oleic, linoleic and stearic acids, polyphenols, and flavonoids (Tan et al. 2019).

Data on the digestive parameters and fecal microbial composition of dogs have also suggested that BSG can be used as a source of fiber in the canine diet (Eisenhauer et al. 2019). Also, recently, BSG was used as a substrate to rear different insects, such as the black soldier fly larva (*Hermetia illucens*) and mealworm larva (*Tenebrio molitor*) (Mancini et al. 2019; Melis et al. 2019; Shumo et al. 2019). These studies have confirmed that it is possible to take advantage of BSG to produce a nutrient-rich feed derived from the black soldier fly larva (Mancini et al. 2019; Melis et al. 2019; Shumo et al. 2019). Microbiological analyses of the larvae reared on the different substrates indicated that BSG administration resulted in decreases in *Staphylococci*, yeast, and mold, and the absence of bacterial endospores (Mancini et al. 2019).

## Brewers’ spent grain in agriculture

Brewers’ spent grain has valuable applications in agriculture. It has been proposed as a promising substrate for production of biocontrol agents, such as the entomopathogenic fungus *Beauveria bassiana*, which is active against *Galleria mellonella* (Qiu et al. 2019). In particular, the high content of starch and fiber in BSG facilitates the germination and mycelial growth of *B. bassiana* conidia. Moreover, *B. bassiana* produced metabolites that promote plant growth (Qiu et al. 2019).

Brewers’ spent grain has direct applications for the soil. Here, BSG increases organic substances, stability of aggregates, water retention, and available water, and also lowers the C/N ratio, which favors mineralization (Aboukila et al. 2018; Mbagwu and Ekwealor 1990). Addition of BSG for an earthworm diet produced enriched soil in Paenibacillaceae, Enterobacteriaceae, Chitinophagaceae, and Comamonadaceae (Budroni et al. 2020). In particular, the low pH and high organic carbon content of BSG were associated with higher abundance of bacterial taxa involved in cellulose degradation and showed high assimilation of ammonia and nitrates. When applied to bioremediation of soil polluted by engine oil, BSG enhanced plant growth and encouraged microbial degradation of hydrocarbons, using bacteria such as *Acinetobacter* spp., *Micrococcus* spp., *Pseudomonas* spp., *Nocardia* spp., and *Bacillus* spp. (Abioye et al. 2010). In particular, with the addition of BSG, the counts of hydrocarbon-using bacteria in the soil were about 5% higher than those without BSG addition.

Brewers’ spent grain was recently used for vermicomposting by the earthworm *Eisenia fetida*, to produce a biological fertilizer (Saba et al. 2019). The vermicompost obtained from BSG respected the parameters of the safety laws: *E. coli* and *Salmonella* spp. were absent in 25 g of the vermicompost, and mycotoxins were degraded during its formation, with ochratoxin A levels below the legal limits. The enzymatic activities revealed a strict link between the microbiota and the quality of the BSG vermicompost, as the higher abundance of fungi and yeast in the BSG vermicompost was accompanied by increased dehydrogenase activity.

## Safety issues

As BSG has been proposed as a component of human and animal nutrition, and also plant nutrition, it is necessary to consider some of the microbiological parameters that influence the quality of this raw material, such as the presence of pathogenic microorganisms and mycotoxins.

### Pathogenic microorganisms

Most potential contaminants of beer originate from the raw materials and/or unclean brewing equipment. The raw materials used in brewing include malt, hops, and occasionally brewing water, and these can be contaminated by microorganisms that must be killed during the brewing process, to prevent wort and beer spoilage (Hill 2009). Correct hygienic standards and regular maintenance must be observed, so that the entire beer production process is not microbiologically compromised (Obi 2017). Water must be free of pathogenic microorganisms, such as coliform bacteria, *E. coli*, *Aerobacter aerogenes*, *Salmonella/Shigella* spp., and *Vibrio cholerae* (Hill 2009). However, to date, no known human pathogens have been found to survive in BSG after the brewing process.

### Mycotoxins

Mycotoxins are secondary metabolites that are produced by fungi and that can affect human and animal health, due to their neurotoxic, immunosuppressive, teratogenic, and carcinogenic effects (Varga et al. 2015). Contamination of food and beverages by mycotoxins is a serious and recurrent problem worldwide, which results in economic losses and health concerns. Several studies have highlighted the need to monitor and determine the exact types and amounts of mycotoxins in cereals and their end-products and by-products, to ensure food and feed safety (Mastanjević et al. 2019; Habschied et al. 2014). Therefore, worldwide legal limits of mycotoxins have been established (Tables 3 and 4).

**Table 3:** Worldwide mycotoxin regulations: limits and guideline for unprocessed and processed cereal in food

**Mycotoxin**	**Regulation level (**μg **kg**^**−1**^)*												
	**European Union**		**USA (FDA)**			**Korea**	**Japan**		**China**	**Indonesia**	**Malaysia**	**Brazil**	
	**European Commission (EC) No. 1881/2006** (2006)	**Recommendation 2013/165/EU** (2013)	**Federal Food, Drug, and Cosmetic Act (FFDC Act 409) Compliance Program for Mycotoxins in Foods Sec. 7307.001**			**Codex Alimentarius Commission CAC/RCP 51-2003 Revised 2017 **(2017)	**Food Safety Commission (FSC No. 48, 2003/12/15)** (2003)		**China Food and Drug Administration (CFDA, GB 2761-2017)** (2017)	**Indonesian National Standardization Agency (SNI) 7385-2009**	**Codex Alimentarius Commission CAC/RCP 51-2003 Revised 2017** (2017)	**Brazilian Surveillance Agency (ANVISA) RDC No. 138, 2019** (2019)	
	**Maximum limit**	**Guidance level**	**FDA action level**	**FDA advisory level**	**FDA guidance level**		**Maximum limit**	**Guidance level**	**Maximum limit**	**Maximum limit**	**Maximum limit**	**Maximum limit**	
Aflatoxin B1	2					10	10		5	15	0.1		
Aflatoxin total (B1, B2, G1, G2)	4		20			15				20	5	5	
Ochratoxin A	3					5			5	5	0.5	20	
Deoxynivalenol	500			1000		1000		1000	1000	1000	1000	750	
Zearalenone	50					200			60			400	
Fumonisins (B1, B2)	500				4000	1000				1000		300	
T-2/HT-2		50											

*1 μg/kg = 1 ppb (parts per billion)

**Table 4:** Worldwide mycotoxin regulations: limits and guideline for cereal products for feed and compound feed

**Mycotoxin**	**Regulation level (μg kg**^**−1**^)*													
	**European Union**		**USA (FDA)**			**Korea**	**Japan**		**China**	**Indonesia**	**Malaysia**	**Brazil**		
	**European Commission (EC) No. 1881/2006** (2006)	**Recommendation EU Reg. 576/2006** (2006)	**Compliance Policy Guide (CPG) Sec. 683.100-2019** (2019)			**(CAC CODEX STAN 193-1995) Revised 2017** (2017)	**Minister of Agriculture, Forestry and Fisheries (MAFF) Act on Safety Assurance and Quality Improvement of Feeds (Law No. 35 of 1953 Revised in 2018)** (2018)		**USDA Hygienic Standard for Feeds GB 13078-2017**	**(CAC CODEX STAN 193-1995) Revised 2017** (2017)	**–**	**Brazilian Surveillance Agency (ANVISA) RDC No. 138, 2017** (2017) **MERCOSUR 2003**		
	**Maximum limit**	**Guidance level**	**FDA action level**	**FDA advisory level**	**FDA guidance level**		**Maximum limit**	**Advisory level**	**Maximum limit**	**Maximum limit**		**Guidance level**		
Aflatoxin B1						10	10		20		–			
Aflatoxin total (B1, B2, G1, G2)	5		20					20		50	–	50		
Ochratoxin A		50							100		–			
Deoxynivalenol		2		5000				1000	1000		–			
Zearalenone		500				3000		1000	500		–			
Fumonisin (B1 B2)		5000	2000		5000			4000	50,000		–			
T-2/HT-2		250							500		–			

*1 μg/kg = 1 ppb (parts per billion)

The contamination of malt by mycotoxigenic fungi and mycotoxins can occur throughout the entire brewer production chain, from the field to the malting and brewing (Bianco et al. 2019, 2018; Mastanjević et al. 2018). Mycotoxigenic fungal species that belong to the genera *Aspergillus* spp. (e.g., aflatoxin, ochratoxin A), *Fusarium* spp. (e.g., T-2 toxin, deoxynivalenol, fumonisins), *Penicillium* (e.g., ochratoxin A, patulin), and *Alternaria* spp. (e.g., alternariol) have been frequently isolated from barley kernels, malted barley, and BSG (Gonzalez Pereyra et al. 2011). Consequently, deoxynivalenol, aflatoxins, fumonisins, trichothecenes, ochratoxin A, and zearalenone are the most significant mycotoxins in the beer chain (Pascari et al. 2018; Habler et al. 2017; Bertuzzi et al. 2011).

Although the concentrations of mycotoxins decrease significantly during the production process (e.g., deoxynivalenol, zearalenone) (Piacentini et al. 2019), BSG can be contaminated by fungi after its production, which will result in increased levels of mycotoxins. Members of the genus *Fusarium* have been frequently isolated in high numbers in BSG, including *F. verticilloides* (50%), *F. proliferatum* (25%), *F. equiseti* (12.5%), and *F. oxysporum* (12.5%) (Mastanjević et al. 2019). *Fusarium* spp. produce the large group of mycotoxins known as the trichotecenes. Further, fumonisins B1 and AFB1 have been detected in malt barley and BSG, which appear to be produced during storage (Gonzalez Pereyra et al. 2011; Kensler et al. 2011). Eight mycotoxins were reported to be produced by *F. culmorum* (i.e., fusarenone-X, 3-acetyldeoxynivalenol, diacetoxyscirpenol, T-2, HT-2, deoxynivalenol, nivalenol, zearalenone), and these have been studied during the production of beer and for its various by-products (i.e., wastewater, spent yeast, BSG) (Mastanjević et al. 2018; Habschied et al. 2014). Malting and brewing by-products can also be contaminated by combinations of mycotoxins and multi-toxins (Mastanjević et al. 2019, 2018; Krstanović et al. 2015; Habschied et al. 2014, 2011). Lotaustraline and tryptophol have been reported at high levels in cereals and in yeast starters used for brewing (Mastanjević et al. 2018). Other mycotoxins and multi-toxins that have been detected in malt and brewing by-products include aurofusarin, beauvericin, brevianamide F, chrysogine, culmorine, 5-hydroxyculmorine, 15-hydroxyculmorine, deoxynivalenol, deoxynivalenol-3-glucoside, linamarin, tentoxine, and zearalenone (Mastanjević et al. 2019).

## Conclusions

Brewers’ spent grain is the main by-product of the brewing industry, and it is regularly available in large amounts at a low market price. Moreover, it is an interesting raw material due to its richness in valuable compounds and nutrients, and the availability of processes for its stabilization. These are all key factors for development of a plethora of different applications that span from the bio/technological production of added-value goods, functional foods, and animal feed, to the generation of other goods of interest for the pharmaceutical and agricultural sectors. Accordingly, there is great interest on BSG exploitation, as indicated by the number of patents concerning BSG preservation, use as a source of useful compounds, and bioconversion (Table 2).

A better understanding of the potential of microorganisms as biocatalysts for BSG transformation is essential for its recycling and exploitation, and also with a view to reduction of its carbon footprint under the concept of a circular economy. Therefore, we might see further interesting new BSG-based applications in the future. In particular, given the biotechnological and health importance of the various microbial groups that have been isolated from BSG, it is essential to study the metabolic relationships among the different microbial communities, and their influences on the final transformation of BSG, to exclude, or on the contrary, to favor, these new processes.
